# Correlation between foot type and posture for the elderly

**DOI:** 10.1186/1757-1146-7-S1-A89

**Published:** 2014-04-08

**Authors:** Kyungock Yi, Namhee Kim

**Affiliations:** 1 Human Movement Study, Ewha Womans University, Seoul, Korea, 120750; 2 Hanbuk University, Donduchun,Kyuggido, 473777, Korea

## 

The purpose of this study was to identify the correlation between foot type and posture. Subjects were 49 elderly people from a Catholic Church in the Seoul area. 13 subjects were men, and 36 were women. Questionnaire was used to identify injuries, blood pressure, history and location of pain, wallet carrying habits for men (left or right side). Resting Calcanel Stance Position (RCSP) was used to identify foot type (pes planus, pes rectus, pes cavus), F/F to R/F and three-dimensional pelvic deviation were also measured,grip strength and posture (sagittal plane & coronal plane). SPSS 18 was used for statistical analysis, along x² (with Scheffe for post-hoc), and Kenndall’s tau_b for the correlation.

1. Foot Deformity

a) 65% of test subjects had normal feet. 69% of elderly men and 64% of elderly women were normal.

b) 21% of elderly women had pes cavus.

c) There was a higher incidence of pes cavus and F/F to R/F angle in the right foot compared to the left.

2. Correlation between foot type and posture

There was a correlation between left foot type and both sagittal postural deviation and coronal shoulder deviation. Subjects with left foot pes planus also exhibited a rearward postural deviation and a left downward sloping shoulder deviation.

There were correlation between deviation in the shoulder, shoulder blade, and pelvis. Thus, subjects with one type of deviation are likely to have corresponding deviations in the other two areas.

For men, shoulders and shoulder blades tilted in the same direction as the pelvis: e.g. Right downward sloping shoulders / shoulder blades corresponded to a right downward sloping pelvis.

Men’s pelvis deviated towards their wallet-carrying side in the transverse plane: e.g. men who habitually carried their wallet in their left pocket had a rearward deviation in their left pelvis and vice versa.

There was a negative correlation between grip strength and injury history. Subjects with stronger grip strength had less injuries and vice versa.

There was a positive correlation between injury history and sagittal postural deviation.
